# Health Risks and Benefits of Alcohol Consumption

**Published:** 2000

**Keywords:** AOD (alcohol or other drug) associated consequences, health risk assessment, beneficial vs. adverse drug effect, protective drug effect, AODR (AOD related) mortality, AODE (effects of AOD use, abuse, and dependence) on stress, societal AODR problems, cognition, heart disorder, myocardial ischemia

## Abstract

Alcohol consumption has consequences for the health and well-being of those who drink and, by extension, the lives of those around them. The research reviewed here represents a wide spectrum of approaches to understanding the risks and benefits of alcohol consumption. These research findings can help shape the efforts of communities to reduce the negative consequences of alcohol consumption, assist health practitioners in advising consumers, and help individuals make informed decisions about drinking.

Forty-four percent of the adult U.S. population (age 18 and over) are current drinkers who have consumed at least 12 drinks in the preceding year (Dawson et al. 1995). Although most people who drink do so safely, the minority who consume alcohol heavily produce an impact that ripples outward to encompass their families, friends, and communities. The following statistics give a glimpse of the magnitude of problem drinking:

- Approximately 14 million Americans—7.4 percent of the population—meet the diagnostic criteria for alcohol abuse or alcoholism (Grant et al. 1994).
- More than one-half of American adults have a close family member who has or has had alcoholism (Dawson and Grant 1998).
- Approximately one in four children younger than 18 years old in the United States is exposed to alcohol abuse or alcohol dependence in the family (Grant 2000).

Alcohol consumption has consequences for the health and well-being of those who drink and, by extension, the lives of those around them. The research reviewed here represents a wide spectrum of approaches to understanding the risks and benefits of alcohol consumption. These research findings can help shape the efforts of communities to reduce the negative consequences of alcohol consumption, assist health practitioners in advising consumers, and help individuals make informed decisions about drinking.

## Measuring the Health Risks and Benefits of Alcohol

Over the years, scientists have documented the effects of alcohol on many of the body’s organ systems and its role in the development of a variety of medical problems, including cardiovascular diseases, liver cirrhosis, and fetal abnormalities. Alcohol use and abuse also contribute to injuries, automobile collisions, and violence. Alcohol can markedly affect worker productivity and absenteeism, family interactions, and school performance, and it can kill, directly or indirectly. On the strength of this evidence, the United States and other countries have expended considerable effort throughout this century to develop and refine effective strategies to limit the negative impact of alcohol (Bruun et al. 1975; Edwards et al. 1994).

In the past two decades, however, a growing number of epidemiologic studies have documented an association between alcohol consumption and lower risk for coronary heart disease (CHD), the leading cause of death in many developed countries (Chadwick and Goode 1998; Criqui 1996*a*,*b*; Zakhari 1997). Much remains to be learned about this association, the extent to which it is due specifically to alcohol and not to other associated lifestyle factors, and what the biological mechanisms of such an effect might be.

### Effects on Physical Health

Cardiovascular diseases account for more deaths among Americans than any other group of diseases. Several large prospective studies have reported a reduced risk of death from CHD across a wide range of alcohol consumption levels. These include studies among men in the United Kingdom (Doll et al. 1994), Germany (Keil et al. 1997), Japan (Kitamura et al. 1998), and more than 85,000 U.S. women enrolled in the Nurses’ Health Study (Fuchs et al. 1995). In research studies, definitions of moderate drinking vary. However, in these studies, most, if not all, of the apparent protective effect against CHD was realized at low to moderate levels of alcohol consumption.

Follow-up of another large U.S. survey, the National Health and Nutrition Examination Survey I (Rehm et al. 1997), found that after an average of nearly 15 years of follow-up, the incidence of CHD in men who drank was lower across all levels of consumption than in nondrinkers. Incidence also was reduced among women, but only in those consuming low to moderate levels of alcohol. In fact, an increased risk was observed in women consuming more than 28 drinks per week.

An association between moderate drinking and lower risk for CHD does not necessarily mean that alcohol itself is the cause of the lower risk. For example, a review of population studies indicates that the higher mortality risk among abstainers may be attributable to socioeconomic and employment status, mental health, overall health, and health habits such as smoking, rather than participants’ nonuse of alcohol (Fillmore 1998).

It is also important to note that the apparent benefits of moderate drinking on CHD mortality are offset at higher drinking levels by increased risk of death from other types of heart disease, cancer, liver cirrhosis, and trauma. The U.S. Department of Agriculture (USDA) and the U.S. Department of Health and Human Services (USDHHS), in the U.S. Dietary Guidelines for Americans, have defined moderate drinking as one drink per day or less for women and two or fewer drinks per day for men (USDA 1995). In addition, the NIAAA further recommends that people aged 65 and older limit their consumption of alcohol to one drink per day.

Cerebrovascular disease, in which arteries in the brain are blocked or narrowed, can lead to a sudden, severe disruption of blood supply to the brain, called a stroke. Ischemic stroke, which is by far the predominant type of stroke, results from a blockage of a blood vessel; hemorrhagic stroke is due to rupture of a blood vessel. Alcohol-related hypertension, or high blood pressure, may increase the risk of both forms of stroke. Yet, in people with normal blood pressure, the risk of ischemic stroke may be decreased due to the apparent ability of alcohol to lessen damage to blood vessels due to lipid deposits and to reduce blood clotting. Alcohol’s anticlotting effects, while perhaps decreasing the risk of ischemic stroke, may increase the risk of hemorrhagic stroke (Hillbom and Juvela 1996). These studies are coming closer to providing a clear picture of the relationship between alcohol and risk of stroke.

The relationship between alcohol consumption and stroke risk has been examined in two recent overviews. In a meta-analysis, researchers compared the relationship between alcohol consumption and the risk of ischemic and hemorrhagic strokes (English et al. 1995). They detected no differences in the risk patterns for the two types of stroke, but found clear evidence that heavy drinking was associated with increased stroke risk, particularly in women.

In contrast, the Cancer Prevention Study II found that, in men, all levels of drinking were associated with a significant decrease in the risk of stroke death, but in women, the decreased risk was significant only among those consuming one drink or less daily (Thun et al. 1997). A recent study reported that among male physicians in the Physicians’ Health Study, those who consumed more than one drink a week had a reduced overall risk of stroke compared with participants who had less than one drink per week (Berger et al. 1999).

Among young people, long-term heavy alcohol consumption has been identified as an important risk factor for stroke (You et al. 1997). Very recent alcohol drinking, particularly drinking to intoxication, has been found to be associated with a significant increase in the risk of ischemic stroke in both men and women aged 16 through 40 years (Hillbom et al. 1995).

The relationship between alcohol consumption and blood pressure is noteworthy because hypertension is a major risk factor for stroke as well as for CHD. A national consensus panel in Canada recently conducted an extensive review of the evidence concerning this relationship (Campbell et al. 1999), concluding that studies have consistently observed an association between heavy alcohol consumption and increased blood pressure in both men and women. However, in many studies comparing lower levels of alcohol use with abstention, findings are mixed. Some studies have found low alcohol consumption to have no effect on blood pressure or to result in a small reduction, while in other studies blood pressure levels increased as alcohol consumption increased.

The possibility that alcohol may protect against CHD has led researchers to hypothesize that alcohol may protect against peripheral vascular disease, a condition in which blood flow to the extremities is impaired due to narrowing of the blood vessels. In a 1985 analysis of data from the Framingham Heart Study, alcohol was not found to have a significant relationship, either harmful or protective, with peripheral vascular disease (Kannel and McGee 1985). However, an important recent study produced different results. In an analysis of the 11-year follow-up data from more than 22,000 men enrolled in the Physicians’ Health Study, researchers found that daily drinkers who consumed seven or more drinks per week had a 26-percent reduction in risk of peripheral vascular disease (Camargo et al. 1997).

Two other studies found inconsistent results with regard to gender. One study of middle-aged and older men and women in Scotland showed that as alcohol consumption increased, the prevalence of peripheral vascular disease declined in men but not in women (Jepson et al. 1995). In contrast, among people with non-insulin-dependent diabetes, alcohol was associated with a lower prevalence of peripheral vascular disease in women but not in men (Mingardi et al. 1997).

There is no question that alcohol abuse contributes significantly to liver-related morbidity (illness) and mortality in the United States. The effects of alcohol on the liver include inflammation (alcoholic hepatitis) and cirrhosis (progressive liver scarring). The risk for liver disease is related to how much a person drinks: the risk is low at low levels of alcohol consumption but increases steeply with higher levels of consumption (Edwards et al. 1994). Gender also may play a role in the development of alcohol-induced liver damage. Some evidence indicates that women are more susceptible than men to the cumulative effects of alcohol on the liver (Becker et al. 1996; Gavaler and Arria 1995; Hisatomi et al. 1997; Naveau et al. 1997).

**Definitions Related to Drinking**

Studies investigating the health effects of alcohol vary in their definitions of “low,” “moderate,” and “heavy” drinking. According to the *Dietary Guidelines for Americans*, issued jointly by the U.S. Department of Agriculture (USDA) and the U.S. Department of Health and Human Services (USDHHS), moderate drinking is no more than two standard drinks per day for men and no more than one per day for women (USDA and USDHHS 1995). The National Institute on Alcohol Abuse and Alcoholism further recommends that people aged 65 and older limit their consumption of alcohol to one drink per day. Information on drinking levels as they are defined in the individual studies cited in this issue can be found in the original references.

**How Much Is a Drink?**

In the United States, a drink is considered to be 0.5 ounces (oz) or 15 grams of alcohol, which is equivalent to 12 oz (355 milliliters [mL]) of beer, 5 oz (148 mL) of wine, or 1.5 oz (44 mL) of 80-proof distilled spirits.

**Does Abstaining Increase Risk?**

Epidemiologic evidence has shown that people who drink alcohol heavily are at increased risk for a number of health problems. But some studies described in this section suggest that individuals who abstain from using alcohol also may be at greater risk for a variety of conditions or outcomes, particularly coronary heart disease, than persons who consume small to moderate amounts of alcohol.

This type of relationship may be expressed as a J-shaped or U-shaped curve, which means that the risk of a disease outcome from low to moderate drinking is less than the risk for either abstinence or heavier drinking, producing a curve in the shape of the letter J or U (see figure).

By examining the lifestyle characteristics of people who consume either no alcohol or varying amounts of alcohol, researchers may uncover other factors that might account for different health outcomes. For example, gender, age, education, physical fitness, diet, and social involvement are among the factors that may be taken into account in determining relative risk of disease.

Similarly, people may quit drinking because of health problems, or even if that is not the case, former drinkers may have characteristics that contribute to their higher mortality risk, such as smoking, drug use, and lower socioeconomic status. If former drinkers are included in the abstainers group, they may make alcohol appear to be more beneficial than it is. Therefore the best research studies will distinguish between former drinkers and those who have never used alcohol.

**Figure:**
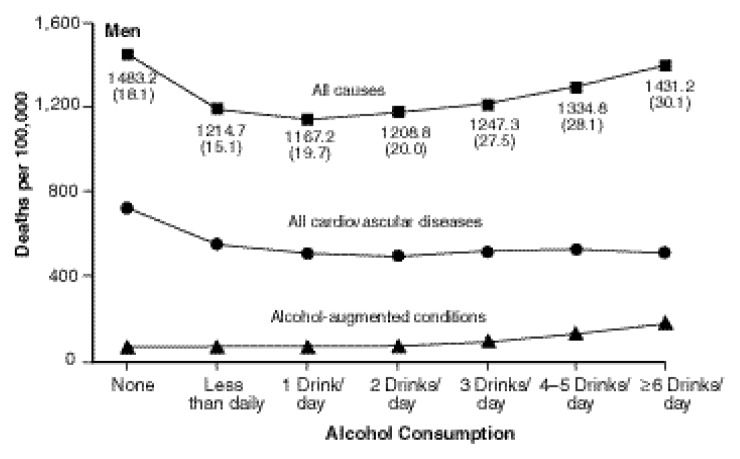
Rates of death from all causes, all cardiovascular diseases, and alcohol-augmented conditions from 1982 to 1991, according to base-line alcohol consumption. SOURCE: Thun et al. 1997. Reprinted with permission from *New England Journal of Medicine*, Vol. 337, pp. 1705–1714, 1997. Copyright 1997, Massachusetts Medical Society. Waltham, MA. All rights reserved.

ReferencesU.S. Department of Agriculture and U.S. Department of Health and Human ServicesHome and Garden Bulletin No. 2324th ed.Washington, DCU.S. Department of Agriculture1995

Alcohol has been linked to a number of cancers, including cancers of the head and neck (mouth, pharynx, larynx, and esophagus), digestive tract (stomach, colon, and rectum) and breast (World Cancer Research Fund/American Institute for Cancer Research [WCRF/AICR] 1997; Doll et al. 1993; International Agency for Research on Cancer [IARC] 1988).

Alcohol is clearly established as a cause of cancer of various tissues in the airway and digestive tract, including the mouth, pharynx, larynx, and esophagus (Doll et al. 1993; IARC 1988; La Vecchia and Negri 1989; Seitz and Pöschl 1997; WCRF/AICR 1997). An increased risk of gastric or stomach cancer among alcohol drinkers has been identified in several, but not the majority, of case-control or cohort studies. The link between alcohol use and chronic gastritis (stomach inflammation) is clear, although progression from chronic gastritis to neoplasia is less well understood and probably involves other factors in addition to alcohol (Bode and Bode 1992, 1997).

In addition, a link between alcohol and breast cancer has been suspected for two decades but the nature of this association remains unclear. (For a more detailed discussion of the role of alcohol in breast cancer, see the article in this issue on medical consequences pp 27–31.)

### Psychosocial Consequences and Cognitive Effects

Alcohol use plays a role in many social activities, from the “business lunch” and parties to special occasions. The benefits to those who drink during social occasions are greatly influenced by culture, the setting in which drinking occurs, and expectations about alcohol’s effects (Goldman et al. 1987; Heath 1987; Leigh 1989; Leigh and Stacy 1991). Stress reduction, mood elevation, increased sociability, and relaxation are the most commonly reported psychosocial benefits of drinking alcohol (Baum-Baicker 1985; Hauge and Irgens-Jensen 1990; Leigh and Stacy 1991; Mäkelä and Mustonen 1988).

There is extensive evidence indicating that people who suffer psychological distress and rely on alcohol to relieve their stress are more likely to develop alcohol abuse and dependence (Castaneda and Cushman 1989; Kessler et al. 1996, 1997). Because vulnerability to alcohol dependence varies greatly among individuals, it is difficult to assess the risk of dependence in relation to how much a person drinks. Two persons exposed to alcohol in exactly the same way may or may not have the same outcome for many reasons, including genetic differences, personality, behavioral features, and environment.

Most mental disorders occur much more often than expected by chance among people who are abusing alcohol or are alcohol dependent (Kessler et al. 1996). Of these individuals, those who are alcohol dependent are more likely than alcohol abusers to have mental disorders. In fact, alcohol dependence elevates the risk for all types of affective and anxiety disorders (Kessler et al. 1996).

Although the relationship between heavy alcohol consumption and cognitive impairment is well established, the effects of moderate drinking on the ability to perform cognitive tasks, including remembering, reasoning, and thinking, are largely unexplored.

Most studies of the relationship between alcohol consumption and other forms of dementia, notably Alzheimer’s disease (Tyas 1996), have failed to find statistically significant associations. However, several recent studies suggest that moderate alcohol consumption may have a positive effect on cognitive function. In an analysis of baseline data (data collected at the beginning of a study) for persons aged 59 through 71 who were enrolled in the Epidemiology of Vascular Aging Study in France, moderate alcohol consumption was associated with higher cognitive functioning among women but not men after a number of possible confounding variables were controlled for (Dufouil et al. 1997). Another study, which followed 3,777 community residents in France who drank primarily wine, found a markedly reduced risk of the incidence of dementia among moderate drinkers relative to abstainers (Orgogozo et al. 1997).

## Effects on Society

Researchers have identified and classified a wide variety of adverse consequences for people who drink and their families, friends, co-workers, and others they encounter (Edwards et al. 1994; Harford et al. 1991; Hilton 1991*a*,*b*). Alcohol-related problems include economic losses resulting from time off work owing to alcohol-related illness and injury, disruption of family and social relationships, emotional problems, impact on perceived health, violence and aggression, and legal problems.

The risk of such consequences for the individual varies widely and depends on the situation. However, researchers have found a general trend toward an increased risk of adverse effects on society as the average alcohol intake among individuals increases (Mäkelä and Mustonen 1988; Mäkelä and Simpura 1985).

Alcohol use is associated with increased risk of injury in a wide variety of circumstances, including automobile crashes, falls, and fires (Cherpitel 1992; Freedland et al. 1993; Hingson and Howland 1993; Hurst et al. 1994). Research shows that as people drink increasing quantities of alcohol, their risk of injury increases steadily and the risk begins to rise at relatively low levels of consumption (Cherpitel et al. 1995). An analysis of risk in relation to alcohol use in the hours leading up to an injury has suggested that the amount of alcohol consumed during the 6 hours prior to injury is related directly to the likelihood of injury occurrence (Vinson et al. 1995). The evidence showed a dose-response relationship between intake and injury risk and found no level of drinking to be without risk.

Patterns of alcohol consumption also increase the risk of violence and the likelihood that aggressive behavior will escalate (Cherpitel 1994; Martin 1992; Martin and Bachman 1997; Norton and Morgan 1989; Zhang et al. 1997). Alcohol appears to interact with personality characteristics, such as impulsiveness and other factors related to a personal propensity for violence (Lang 1993; Zhang et al. 1997). Violence-related trauma also appears to be more closely linked to alcohol dependence symptoms than to other types of alcohol-related injury (Cherpitel 1997).

Patterns of moderate drinking, on the other hand, have been associated with a key health benefit—that is, a lower CHD risk. Research is now in progress to clarify the extent to which alcohol itself, or other factors or surrogates such as lifestyle, diet, exercise, or additives to alcoholic beverages, may be responsible for the lower risk. Broader means of quantifying the relationships between relative risks and specific consumption levels and patterns are needed to describe epidemiologic findings more clearly and simply, and translate them into improved public health strategies.

## The Overall Impact

The overall impact of alcohol consumption on mortality can be assessed in two ways (Rehm and Bondy 1998): (1) by conducting meta-analyses using epidemiologic studies that examine all factors contributing to mortality, or (2) by combining risk for various alcohol-caused diseases with a weighted prevalence or incidence of each respective disease.

The meta-analysis approach to assessing overall mortality was used by researchers to examine the results of 16 studies, 10 of which were conducted in the United States (English et al. 1995). In this overview, researchers found the relationship between alcohol intake and mortality for both men and women to be J-shaped curves: the lowest observed risk for overall mortality was associated with an average of 10 grams of alcohol (less than one drink) per day for men and less for women. An average intake of 20 grams (between one and two drinks) per day for women was associated with a significantly increased risk of death compared with abstainers. The risk for women continued to rise with increased consumption and was 50 percent higher among those consuming an average of 40 grams of alcohol (between three and four drinks) per day than among abstainers. Men who averaged 30 grams of alcohol (two drinks) per day had the same mortality as abstainers, whereas a significant increase in mortality was found for those consuming at least 40 grams of alcohol per day.

The proposed J-shaped relationship between alcohol intake and mortality does not apply in all cases, however. For example, because most of the physiologic benefit of moderate drinking is confined to ischemic cardiovascular conditions, such as CHD, in areas of the world where there is little mortality from cardiovascular diseases, alcohol provides little or no reduction in overall mortality. Rather, the relationship between intake and all-cause mortality assumes more of a direct, linear shape (Murray and Lopez 1996*c*), with increasing consumption associated with higher overall mortality. The same holds true for people under age 45, who have little ischemic cardiovascular mortality (Andréasson et al. 1988, 1991; Rehm and Sempos 1995).

Quantifying the level of disability and morbidity related to alcohol can be difficult, in large part because few standardized measures exist. One way to quantify the relationship between alcohol and health-related consequences is to use a measure called the disability-adjusted life year (DALY), which may prove useful in summarizing the effects of alcohol on the full spectrum of health outcomes.

In the Global Burden of Disease Study (Murray and Lopez, 1996, 1997*b*), the researchers combined years of life lost and years lived with disability into a single indicator, DALY, in which each year lived with a disability was adjusted according to the severity of the disability (Murray and Lopez 1997*b*,*c*). The study found tremendous differences in alcohol’s impact on disability across different regions of the world. The most pronounced overall effect was observed in established market economies. The researchers found the smallest effect of alcohol in the Middle Eastern crescent, which is not surprising given the region’s high proportion of abstinent Islamic populations (Murray and Lopez 1997*a*).

Epidemiologic studies have long provided evidence of the harm alcohol can cause to individual health and to society as a whole. Newer studies have identified an association between low to moderate alcohol consumption and reduced CHD risk and overall mortality. The most significant association with lower CHD risk is largely confined to middle-aged and older individuals in industrialized countries with high rates of cardiovascular diseases. Elucidation of the mechanisms by which alcohol affects CHD risk will clarify the relationship and may enable scientists to develop pharmacologic agents that could mimic or facilitate the positive effect of alcohol on health (Hennekens 1996; UK Inter-Departmental Working Group 1995; USDA 1995). At this point, research clearly indicates that no pattern of drinking is without risks. However, for individuals who continue to consume alcohol, certain drinking patterns may help reduce these risks considerably.

Among teenagers and young adults in particular, the risks of alcohol use outweigh any benefits that may accrue later in life, since alcohol abuse and dependence and alcohol-related violent behavior and injuries are all too common in young people and are not easily predicted. To determine the likely net outcome of alcohol consumption, the probable risks and benefits for each drinker must be carefully weighed.
